# Live Long and Prosper: Potentials of Low-Cost Consumer Devices for the Prevention of Cardiovascular Diseases

**DOI:** 10.2196/med20.2667

**Published:** 2013-08-12

**Authors:** Jochen Meyer, Andreas Hein

**Affiliations:** ^1^R&D Division HealthOFFIS Institute for Information TechnologyOldenburgGermany; ^2^School of Medicine and Health SciencesCarl von Ossietzky University OldenburgOldenburgGermany

**Keywords:** primary prevention, cardiovascular diseases, user-computer interface

## Abstract

**Background:**

Cardiovascular diseases (CVD) are one of the major causes of death worldwide. Personal behavior such as physical activity considerably influences the risk of incurring a CVD. In the last years numerous products such as pedometers have become available on the mass market that allow monitoring relevant behaviors and vital parameters. These devices are sufficiently precise, affordable, and easy to use. While today they are mostly lifestyle oriented they also have considerable potential for health and prevention.

**Objective:**

Our goal is to investigate how recent low-cost devices can be used in real-life settings for the prevention of CVD, and whether using these devices has an advantage over subjective self-assessment. We also examine whether it is feasible to use multiple of such devices in parallel.

**Methods:**

We observe whether and how persons are willing and able to use multiple devices in their daily lives. We compare the devices’ measurements with subjective self-assessment. We make use of existing low-cost consumer devices to monitor a user's behavior. By mapping the devices' features with pre-defined prevention goals we ensure that the system collects meaningful data that can be used to monitor the individual's behavior. We conducted a user study with 10 healthy adults to measure usability and to identify problems with sensor use in real life. The participants used the devices' original portals to monitor their behavior. The subjects (age range 35-75) used an off-the-shelf pedometer and a sports watch for 4 weeks.

**Results:**

The participants responded in principle positively to the use of the devices. Analyzing the sensor data, we found that the users had some difficulties in operating the devices. We also found that the participants' self-assessment of their health behavior was too optimistic compared to the monitored data. They rated the usability of the overall system with 71 of up to 100 points in the "System Usability Scale".

**Conclusions:**

Our study indicates that today's devices are suitable for a long term monitoring of health for the prevention of CVD. Using the devices provides more precise data than a subjective self-assessment. However usability and acceptance of the systems are still major topics.

##  Introduction

### Background

Cardiovascular diseases (CVD) are the number one reason of death globally [1]. To a large extent they are caused by behavioral risk factors such as lack of physical activity or an unhealthy diet. A healthy lifestyle is a life-long effort that involves multiple facets such as daily activity, fitness, sleep, and many more. Monitoring is one of the key technologies of many persuasive health systems [2,3]. While many devices for monitoring one or more of these behaviors exist on the market already, it seems unlikely that the universal device that monitors everything and is liked by everybody will ever be available. Hence, a person who would wish to use a pervasive system for the prevention of CVD would have to permanently use multiple devices simultaneously.

We therefore investigated whether a person would be able to use multiple commercial off-the-shelf devices for a longer period of time in daily use for a reliable behavior monitoring for the prevention of cardiovascular diseases. We also examined whether the measured data from the devices provide added value over a simple subjective self-assessment. 

### Prevention of Cardiovascular Diseases

#### Overview

Risk factors for incurring CVD include vital parameters such as blood lipids or blood pressure, behaviors such as physical activity or nutrition, and other factors such as environmental factors or psychosocial stress (eg, [1,4,5]). For the scope of this study, we are focusing on 2 aspects that are of major interest to most concerned persons and that may be monitored using consumer devices, namely physical activity and sleep.

#### Physical Activity

All guidelines for heart-friendly lifestyle [5] recommend physical activity as a key behavior. Usually, 2 physiologically different types of activities are recommended, “daily activity” on a moderate level of intensity such as walking or slow cycling, and vigorous or “endurance sports” activity such as jogging or fast cycling. While the detailed specifications and wording may vary, there is a general consent that daily activity should be performed preferably daily for at least 30 minutes, and endurance sports should be performed at least twice a week.

Research [6] also indicates that endurance sport 3 times a week for at least 30 minutes each already achieves the maximum effect for cardiac health. Only if the user misses some of the trainings, he may partially compensate by daily activity. However, the daily activity does not have the same positive effects as a real training, so a lack of training cannot fully be compensated by daily activity. Moreover, in order to be effective for the heart at all, daily activity must happen in intervals of at least 10 minutes without a break [7]. With 30 minutes of activity each day of the week, the maximum effect has been reached. A lack of activity on one day cannot be compensated by more activity on subsequent days.

#### Sleep

Although sleep is not as unanimously part of the guidelines, there is a growing body of evidence [8] that sleep behavior has a major effect on cardiac health. A meta-study [9] has shown that people sleeping 6-8 hours a night have no increased risk of long-term health consequences, but people consistently sleeping 5h or less should be regarded as higher risk group for cardiovascular morbidity. On the other hand, sleeping 9h or more per night may be an indication for subclinical or undiagnosed co-morbidity. Home monitoring one’s sleep behavior may help to increase awareness for a good sleep behavior and to identify potential problems [10] and is therefore potentially a feasible tool for the prevention of CVD.

### Pervasive Systems for Health Monitoring

With the availability of appropriate sensors and devices, in the last 10 years many systems have been developed that use monitoring as a basic technology in systems that aim to support a healthy lifestyle. Early such systems put a straightforward and actionable link between monitored data on the one hand and envisaged behavior or outcome on the other. Physical activity as a frequent example is measured using pedometers to count the number of steps, and the envisaged behavior is to achieve a minimum number of steps each day. Fish’n Steps [11] or Chick Clique [12] are examples that combine a playful interface and elements of competition. The UbiFitGarden system [13] was shown to induce an envisaged health behavior also over a period of several months. There is increasing evidence that mobile interventions are generally effective to encourage physical activity [14]. Such types of interventions have reached the market with sensor-based systems such as Fitbit or Nike FuelBand for daily activity, or Zeo for sleep.

Research is now addressing systems for more complex health questions that require monitoring multiple behaviors and need a more complex data analysis to identify health states and outcomes. Monarca [15] uses an interactive application and various sensing devices for identification of episodes in the treatment of bipolar disorder, based e.g. on a sentiment analysis of text messages, and on the frequency of phone calls. JogFalls [16] combines activity monitoring, diet logging, and monitoring of certain vital parameters for the management of diabetes. Such systems clearly have a high potential for the management of chronic diseases, also because they fulfill the user’s need to go beyond mere presentation of data [17]. However, by design they are not intended for everyday use by healthy persons aiming to stay healthy: They require interaction and use of specific, potentially obtrusive sensors. Mobile phones clearly are an important enabling platform [18], however they cannot be considered a universal solution.

As persuasive technology for prevention and well-being becomes more and more an aspect of our daily life, usability, user acceptance, and suitability for everyday use are increasingly important. In a workshop conducted at PervasiveHealth 2012 [19] we identified major points that distinguish the preventive use of persuasive health technologies from management of diseases: People will be using multiple and different devices; preventive systems are used over a long period of time, potentially over decades or even life long; people have complex goals that cannot easily be broken down into daily advice.

### Rationale and Goal

The goal of our study is to identify user requirements for the use of multiple devices as part of a system on the prevention of cardiovascular diseases. Particularly we want to understand if the users are able to interact with different devices at the same time, if they would be able to interpret the gathered data, if these devices can be helpful for sustaining a healthy lifestyle, and if they can improve the self-assessment. We furthermore wanted to examine whether the effort of sensor-based monitoring has advantages over subjective self-assessment.

Therefore we conducted a 3 week study with 10 participants that used a small set of sensors to monitor multiple behaviors that contribute to a heart-friendly lifestyle.

##  Methods

### Set-Up of the Study

We focused on 2 factors of heart-friendly living, physical activity and sleep. Physical activity is further broken down into daily activity and endurance training. To monitor these 3 behaviors, the participants received 2 different devices: A Fitbit Ultra pedometer, and a Garmin Forerunner 110 training watch. We chose these devices as they are widely available and prototypical representatives of products for monitoring personal activities. With prices ranging from about 50-150 Euro they are not particularly cheap but affordable for many persons. We decided not to use any of the available smartphone apps collecting this data since - in spite of their increasing popularity—the majority of people still do not own a smartphone that is powerful enough for monitoring. Moreover as a universal platform that is not tailored to the specific needs of collecting long-term activity data smartphones still face a number of issues including battery runtime and obtrusiveness of wearing the device.

The Fitbit Ultra is a lightweight electronic pedometer that may be worn in the pocket or attached to the clothes. It counts the steps taken per minute and transfers the data wirelessly and without interaction via a docking station on a local PC to the online platform fitbit.com. The Fitbit may also be used to monitor sleep. For the study we used sleep duration by manually marking start and end of the night by pressing a button on the device.

The Garmin Forerunner 110 is a sports watch with a breast belt to monitor the heart rate and an integrated GPS for monitoring the pace during the workout. The watch may also be used indoors without GPS, or it may be used without the heart rate belt. The watch may be connected to a PC using a special USB cable for uploading the data to a dedicated software or an online portal via a browser plug-in. We used the runkeeper portal to collect the user’s endurance training data.

With these devices we collect the following data:

- the step count of the user for each minute of the day
- the start time and duration of an endurance training
- the start and end time of sleep, as manually marked by the user

More detailed data is available in the portals but not used within the study.

### Participants

Participants of our study were 10 subjects, 5 female, and 5 male, who were customers of a medically oriented gym. The age ranged from age class 35-44 to 65-74, average age was 54 (SD 12 years). In the group were 2 couples. All subjects already used a body scale (digital or analogue), 3 used a blood pressure monitor, one had used a pedometer and 3 had used a sports watch with heart rate monitor before. All subjects were under regular supervision of the gym’s physician. One participant had a previous cardiac condition.

All participants used a PC and the Internet fairly frequently, but had no particular interest in new technologies. They were very interested in healthy living and were doing sports regularly. They felt they had a fairly good knowledge about healthy living and a good self-assessment of their behavior. The main reason for participation was to learn about one’s own health behavior, other reasons were interest in new devices and intended health behavior change.

We piloted the system before with 3 persons who tested the system for one week each.

### Conductance of the Study

The study took place during 3 weeks in November 2012 in North-West Germany. We had one kick-off meeting where we explained the study and instructed the participants in detail how to set up and use the devices as well as one closing meeting. In between, we contacted the users by phone or by email to solve potential issues.

The subjects’ mission for the study was to follow our selected guidelines for heart friendly living throughout the study: “Do a fitness training of at least 30 minutes at least 3 times a week. If that is not possible be active for at least 30 minutes each day in intervals of at least 10 minutes each. Sleep between 6 and 8 hours each night. Monitor and regularly review your behavior using the devices and systems.”

In the closing meeting, all participants filled out a 22-item questionnaire: The first section was the German version of the system usability scale (SUS). The next section asked for experiences using the system and for effects on the participants’ health behavior. We also asked for subjective self-assessment on how well they followed guidelines given. Possible answers ranged from “Perfect” (4 points) up to “not at all” (0 points). The questions in this section were adapted from the IPAQ questionnaire [20]. The last section of the questionnaire asked for potential future extension of the system. We discussed the participants’ experiences using the devices and portals and also the emerging difficulties they experienced.

## Results

### Data Collected

The 10 participants used the devices and collected data for an overall period of 225 days and 219 nights (see Multimedia Appendix 1).

On 224 days (99.55%) step data was collected using the Fitbit. The average number of steps per day was 10,045 (SD 3243, minimum average per person 4885, maximum 14,918).

The active minutes per day were estimated based on the minute-wise step data of the Fitbit: When for 10 minutes the step-count per minute was above a minimum threshold of 40 steps we would assume this to be an active interval. One minute with a lower value in between was allowed to reflect, for example, necessary traffic light stops when walking through a city. We chose the threshold based on our own experiments. According to our internal experiments the resulting assumptions are a fair estimation. The average active minutes per day were 41.4 (SD 22.2, minimum average per person 9.92, maximum 68.85).

The participants altogether recorded 45 endurance training sets. All of them were at least 30 minutes. Twelve (27%) had a duration of 30-60 minutes, 14 (31%) 1-2 hours. 10 (22%) had a duration of more than 12 hours.

For 180 nights (80%) sleep data was collected. 83 of the sleep records (46%) had a duration of 6-8 hours, 51 (28%) 8-9 hours, 6 (3%) 5-6 hours. 14 (8%) had a duration of more than 16 hours.

The participants’ self-assessments on how well they followed the recommendations for daily activity, fitness trainings and sleep is shown in Table 1 below.

### Qualitative Experiences From Using the Sensors

Setting up the devices mostly worked flawlessly, but some problems still were reported. Some participants had technical difficulties in the installation process. Some of the devices were faulty and had to be exchanged. Non-standard installations raised further questions. The concept of the local installation of the Fitbit service component in combination with the Fitbit portal was difficult to understand and caused confusion when one participant switched to a new PC at home.

In daily use, most participants were excited about the Fitbit for monitoring their daily activity. They very much appreciated the feedback on their daily activities and were sometimes quite surprised about the results in comparison to their self-assessment. The preferred level of detail of the data was quite heterogeneous: While some participants were very interested to understand the details and reviewed the data in the portal very carefully, others were happy just to see the number of steps per day on the device’s display, and hardly looked into the portal. All in all, the participants found the Fitbit device very easy and intuitive to use, whereas the portal was considered more complex and difficult to use.

A number of negative points of the Fitbit were also discussed. The device was occasionally forgotten. Cycling as part of the daily activities was not well accounted for in terms of step count. Not everybody liked to keep the Fitbit in their pocket or clipped to the clothes. Particularly women said that the clothes they wear may not have pockets to store the device. Some participants forgot to take the Fitbit off their pocket when changing clothes throughout the day. Several persons suggested having, for example, a bracelet rather than a clip.

Although prior pilot tests had indicated that sleep behavior might be an issue of interest, most participants were in the end not particularly interested. They said they tended to forget to mark their sleep or wake-up times, and they did not get any further insights from it.

The feedback on the Garmin Forerunner was less positive. There were difficulties in getting the device to work. The general concepts of GPS monitoring and heart rate monitoring were not well understood. The required delay for getting a GPS fix outdoors was considered annoying. When used indoors the lack of GPS caused considerable confusion. In general, especially indoors, little added value was seen using the watch for heart rate monitoring. People liked using the watch rather outdoors. They would not just use it for monitoring the actual endurance trainings such as walking, but also take into account other activity such as their daily cycling or an occasional hiking tour.

Uploading the training data from the watch to the Runkeeper portal was considered to be slightly annoying, but in the end worked for most participants. However, the portal was not appreciated well. There was little advantage seen for entering the data into the system, and the results were only rarely used. The participants commented that they would in principle be interested to monitor their training, but that monitoring should be much better connected to their situation, for example, by directly linking it to their gym visits.

**Table 1:** Self-assessment of activities versus monitored behavior.

Participant	Daily activity self-assessed	Daily activity actual level by minutes	Actual activity level by steps	Fitness activity self-assessed	Fitness activity actual level	Sleep self-assessed	Sleep actual level
1	3	1.51	1.15	3	1.61	4	2.09
2	-	3.60	3.41	4	2.00	1	2.35
3	4	3.09	3.01	3	1.58	4	1.74
4	4	2.73	2.63	4	3.00	4	3.03
5	3	0.99	1.11	2	2.16	4	1.52
6	3	2.90	3.04	2	0.00	1	2.47
7	1	3.13	3.73	4	3.00	4	3.81
8	2	2.06	2.47	2	2.33	4	2.23
9	3	2.96	2.83	0	0.74	4	2.89
10	4	3.43	3.45	4	2.79	2	3.06

### Usability and Users’ Experiences

We asked the users for the usability of the overall system, for possible effects of the system on their health behavior, and for suggestions for future improvements.

The usability was measured using the German version of the System Usability Scale [21]. The average score is 71 (SD 17.7).

Most participants said that the devices helped them to better understand their own behavior and motivated them to a healthier lifestyle. The participants felt that using the system increased the awareness towards their personal activity. Many participants planned their activities more carefully. Some participants also felt that during the study they learned to better assess their behavior. Therefore they would be able to live healthier afterwards even without using the devices. However, the examples given by the participants were addressing mainly daily activities. Endurance workouts were only occasionally mentioned, and sleeping duration was not an issue at all.

One participant was generally more critical about self-monitoring, seeing also the risk of over-motivation and distorted feedback, since the devices just provide a very selective view on one’s behavior.

Several participants explicitly mentioned that dealing with the multiple platforms was difficult and caused confusion. Therefore they suggested an integrated platform that would allow seeing all the data in one place.

Although all participants were strongly aware of the do’s and don’ts of a healthy lifestyle, the goal to live heart friendly was not appealing to them. They understood the necessities of activity and sleep, but found little motivation in following this goal. Within the scope of the study they were much more eager to see their physical daily activity. Several participants suggested that other goals would be more interesting, including weight control and increasing fitness. They also suggested to include more monitoring options, for example, for weight, nutrition, or blood pressure.

## Discussion

### Sensor Use

The Fitbit was used by all participants virtually every day for monitoring daily activity. We therefore assume that this device is in principle well accepted. However, from the participants’ feedback we also must assume that the device was occasionally not worn during some parts of a day. The step data per day is therefore likely to be incomplete. We conclude that the concept of the Fitbit as an easy to use device is well accepted, but different forms of pedometers such as a bracelet rather than a clip might have resulted in more complete data for some of the participants.

The participants recorded 45 workouts altogether. 14 of these (31%) were above 4 hours duration for 4 participants and 10 of these (22%) even above 16 hours duration for 2 of the participants. Longer durations might be cycling or hiking tours. However, trainings above 16 hours duration can only be explained by the participants forgetting to mark the end of the training.

Recording sleep required pressing a button in the evening and again in the morning. With 80% of the nights covered, sleep data was less complete than the data on daily activity. We therefore assume that occasionally the device was forgotten, or was not used intentionally. From the recorded sleep durations it is noticeable that a number of sleep records are overlong, above 12 hours (5 nights, 3%) or even above 16 hours (14 nights, 8%). Particularly in the latter case we can assume that pressing the button in the morning was forgotten.

### Self Assessment Versus Monitored Behavior

We compared the participants’ self-assessments with the measurements taken by the sensors. The self-assessment took place at the end of the study, after 3 weeks only, so there is likely a recall bias limiting the precision of the data. On the other hand, during the 3 weeks the participants got regular feedback on their actual behavior from the devices, which should increase their awareness for their behavior and mitigate the effects of the recall bias. The results are summarized in Table 1. For each of the 3 behaviors, the columns show from left to right the points as subjectively assessed by the participants, and the actual achieved points as monitored by the devices—for daily activity in 2 different methods. The details are subsequently described.

For daily activities we took into account the active minutes in intervals of 10 minutes each. We scaled this into a 0-4 scale from 0 (no active minutes) to 4 (30 minutes or more of activity). The participants had reported that they observed their daily step count with high interest, so we could expect quite a good estimation. However, 5 participants were too optimistic in their self-assessment, 3 were quite precise, and one was too pessimistic. One participant did not assess his activity.

We also used the daily step count sum as an alternative measurement to mitigate potential weaknesses of our approach for estimating the active daily minutes. By adopting the Tudor-Locke scale [22] we gave 0 points for 0 steps, 1 point for 5000 steps, 2 for 7500, 3 for 10,000 and 4 for 12,500 and more. We linearly interpolated points for step values in between. The resulting scores deviate only slightly from the one based on our own estimation (average deviation 0.04, SD 0.29) and do not change the overall picture.

For training, we counted for each day the number of fitness trainings the participant had recorded for this and the preceding 6 days. All trainings recorded were longer than 30 minutes For each day with at least 3 trainings on that and the preceding 6 days we scored 4 points, for 2 trainings 3 points, for 1 training 2 points and for no training 0 points. We compared this to the participants’ self-assessment. 7 participants were too optimistic in their self-assessment, and 3 were slightly pessimistic.

For sleep, we scored 4 points for each night with 6-8 hours. We scored 0 points for 5 and 9 hours, 4 for 6 and 8 hours, and linearly interpolated values in between. 0 points were scored for nights shorter than 5 and longer than 9 hours. For calculating the participant’s average sleep score, we omitted sleep records of more than 16 hours duration, as we assume a faulty use of the sensor. Compared to the participants’ self-assessment, 6 participants were too optimistic, in 1 case the assessment was about correct, and in 3 cases it was too pessimistic.

Our comparison between self-reported and monitored data is partially in line with other studies. For physical activity [20] concludes that for vigorous activities there is a strong correlation between self-assessment and monitoring, while we found some deviations. For medium activities, [20] finds a fair to moderate correlation, which is quite in line with our observations. Differences could be explained in different self-assessment methods: We used only an ex-post questionnaire after 3 weeks, while [20] used a logbook. For sleep [23] finds that in average the self-assessment is close to the objective measurement, but there are considerable individual differences. This is well reflected by our observations. In general, our participants tend to assess themselves more positively. This might be explained by the fact that our participants had the mission to follow some specific guidelines. Not having succeeded in following these might therefore be considered a failure, so the participants might have had the trend to show themselves in a more positive light.

### Limitations

The study has some limitations. The measured data may be partially wrong due to a non-identified faulty use or because participants didn’t record all activities. Our measurement of active minutes based on analyzing the steps by the minute is not precise. And our mapping of the activities to 0-4 points scale compared to the participants’ self-assessment scale 0-4 may be imperfect. Taking into account the relatively small sample size of 10 persons, the figures as outlined in Table 1 should be understood to underline our qualitative statements, not as quantitative results in themselves.

However, with 10 participants the sample is large enough to gain a better understanding of how people use pervasive health services and what the current problems are. The demographic of the participant group is rather broad with respect to age range and to participation of female and male persons, and it included 2 couples. The participants were in general just average technically skilled. The group was in general healthy and didn’t suffer from a particular disease. The interest in healthy living was probably above average, but not exceedingly high. Therefore we think that our participants are fairly close to the “average target group”

### Design Implications

Our results reveal a number of implications for the future design of systems for the prevention of cardiovascular diseases:

We gave 2 devices to the users, both of which were mass market products and should in principle be easy to install and use. We explained carefully the required procedures to the participants. Nevertheless, installing and operating the devices has shown to be a considerable effort for the users. We found that virtually every possible interaction with the devices required some training and lead to some faulty operations and possible errors in data. However we also found that the participants were quite eager to resolve the issues if their use promised personal advantages. Therefore, while it is basically always a good idea to keep a system as simple as possible, we also find that users are willing to accept some level of complexity provided that the promised advantage is high enough for them. However, faulty use and incomplete data will always happen, and any system using that data will have to cope with that.

Wearing and using the devices was rated differently by the participants. The Garmin watch was mostly liked for outdoor use, but it was too complex for indoor workouts. Also the Fitbit, in spite of its high acceptance, was not the perfect device for all the users. Some participants would have preferred a bracelet over a clip. Prevention devices are used for a longer period of time, so users must have the choice of devices they use, and they might want to use different devices for the same purpose, changing from day to day. A pervasive prevention system must therefore deal with heterogeneous devices and not focus on one or 2 specific products.

Our participants were very interested in healthy living and had probably an above-average degree of knowledge on that issue. The intention to live heart-healthy was generally understood and appreciated. Nevertheless our goal and guidelines were not particularly appealing to them. They found little motivation trying to live more heart-healthy in general, and were much more focusing on increasing daily activity. Moreover, the participants suggested goals such as weight control or increasing fitness. Therefore we conclude that general goals for healthy living must be broken down into concrete and actionable sub-goals that are personalized to match the individual user’s needs and that may well change over time. A preventive system can then assist the user in following these sub-goals and guidelines.

All participants liked getting insights into their own behavior. However, the requested level of detail was different between the users. For the Fitbit some users were happy just seeing the number of daily steps they achieved, whereas others were keen to understand how different types of activities contribute to step counts and active minutes. Sleep duration was in general not considered interesting by the users. However, from pilot tests in slightly different set-ups we found that people may be interested in sleep quality. Therefore we think there is a mutual influence of the choice of devices and the definition of personalized goals. A device’s properties obviously limit the possible level of detail of the monitored data: If the user’s preferred pedometer doesn’t allow monitoring active minutes, activity goals may need to be defined based on daily step counts rather than the more detailed active minutes. The other way around, the user’s goals influence the choice of devices: If sleep quality is important, the user may wish to use a device like the Zeo, whereas for mere sleep duration the Fitbit approach would be fine.

Our participants had decent knowledge about healthy living and followed a healthy lifestyle. Nevertheless, when asked for their subjective self-assessment regarding their behavior during the study, the participants tended to over-estimate their own behavior. Therefore behavior monitoring using technical devices provides a more reliable base data for recommendations on healthy behavior than self-assessment alone.

### Summary and Outlook

We investigated how low-cost monitoring devices can be used in the context of prevention of cardio-vascular diseases. We learned that using devices is a challenge to the user, but users are willing to cope with it if their advantage is clear. However, users prefer different devices therefore we believe that also in the future we will have heterogeneity of devices rather than the one universal product.

There was a tendency that a person’s subjective self-assessment is more optimistic than the data monitored by the devices. Therefore the objective monitoring is potentially better suited for observing and reflecting health behavior than a subjective self-assessment.

We believe that preventive systems in the future will be platforms that integrate multiple data sources to provide the user with a unified view. One main challenge clearly is the analysis and interpretation of this heterogeneous data to infer medically valid conclusions on the user’s health. However this is what we need to turn the existing personal data into personalized knowledge.

## Multimedia Appendix 1

Collected user data on daily activity, sleep and endurance training.

## Abbreviations

CVD: cardiovascular diseases
SUS: system usability scale
